# Evaluation of Serum Calcium as a Predictor of Biochemical Recurrence following Salvage Radiation Therapy for Prostate Cancer

**DOI:** 10.1155/2013/239241

**Published:** 2013-03-31

**Authors:** Jennifer L. Peterson, Steven J. Buskirk, Michael G. Heckman, Alexander S. Parker, Nancy N. Diehl, Katherine S. Tzou, Nitesh N. Paryani, Stephen J. Ko, Larry C. Daugherty, Laura A. Vallow, Thomas M. Pisansky

**Affiliations:** ^1^Department of Radiation Oncology, Mayo Clinic Florida, Jacksonville, FL 32224, USA; ^2^Section of Biostatistics, Mayo Clinic Florida, Jacksonville, FL 32224, USA; ^3^Department of Urology, Mayo Clinic Florida, Jacksonville, FL 32224, USA; ^4^Department of Radiation Oncology, Mayo Clinic Rochester, Rochester, MN 55905, USA

## Abstract

*Background*. Previous reports have shown a positive association between serum calcium level and prostate cancer mortality. However, there is no data regarding whether higher serum calcium levels are associated with increased risk of biochemical recurrence (BCR) following salvage radiation therapy (SRT) for prostate cancer. Herein, we evaluate the association between pretreatment serum calcium levels and BCR in a cohort of men who underwent SRT. *Methods*. We evaluated 165 patients who underwent SRT at our institution. Median dose was 65.0 Gy (range: 54.0–72.4 Gy). We considered serum calcium as both a continuous variable and a 3-level categorical variable (low [≤9.0 mg/dL], moderate [>9.0 mg/dL and ≤9.35 mg/dL], and high [>9.35 mg/dL]) based on sample tertiles. *Results*. We observed no evidence of a linear association between serum calcium and BCR (relative risk (RR): 0.96, *P* = 0.76). Compared to men with low calcium, there was no significantly increased risk of BCR for men with moderate (RR: 0.94, *P* = 0.79) or high (RR: 1.08, *P* = 0.76) serum calcium levels. Adjustment for clinical, pathological, and SRT characteristics in multivariable analyses did not alter these findings. *Conclusion*. Our results provide evidence that pretreatment serum calcium is unlikely to be a useful tool in predicting BCR risk following SRT.

## 1. Introduction

Approximately, one-third of men treated with a radical prostatectomy (RP) for prostate cancer will have biochemical recurrence (BCR) within 10 years, and in two-thirds of these men on active surveillance, metastatic disease develops within 10 years [1]. Salvage external beam radiation therapy (SRT) appears to positively affect this natural history when it is initiated early in the course of postoperative BCR [2–4]. A key clinical issue centers on the need to predict which patients with a detectable serum prostate-specific antigen (PSA) after RP have local recurrence versus micrometastatic disease. Accurate means of distinguishing these two groups of men would allow for better selection of patients as candidates for local SRT.

We developed and published a scoring algorithm based on readily available clinicopathologic features to help predict which men will experience BCR after SRT and thus provide a guide for clinicians when counseling patients [2]. More recently, we evaluated RP specimens for the ability of specific tumor-based biomarkers (e.g., Ki-67 and B7-H3) to predict which men will respond to SRT [5–7]. Based on our reports, information on clinicopathologic features and tumor-based biomarkers can assist in the appropriate selection of men as good candidates for SRT; however, there is still uncertainty regarding which subsets of patients receive the least benefit from SRT. As such, additional prognostic tools are needed to help further stratify patients for greater individualization of prostate cancer treatment.

Previous investigators have reported an association between increased serum calcium levels and prostate cancer mortality [8, 9]. In a prospective cohort of patients from the National Health and Nutrition Examination Survey (NHANES) I and the NHANES Epidemiologic Followup Study [8], men with serum calcium levels in the highest tertile (mean serum calcium, 10.2 ± 0.3 mg/dL) had more than 2.5-fold increased risk of death attributed to prostate cancer. These initial findings were confirmed in an independent, prospective cohort of men from NHANES III [9].

On the basis of the reported association between increased serum calcium and prostate cancer mortality, we hypothesized that men with higher serum calcium levels may be at greater risk of BCR following SRT. Herein, we evaluate the association between pretreatment serum calcium levels and risk of BCR following SRT using a cohort of men who received SRT for a detectable PSA following RP.

## 2. Materials and Methods

### 2.1. Patient Selection and Outcome Definition

With the approval of the Mayo Clinic Institutional Review Board, a retrospective review was performed on all patients who underwent SRT for a detectable PSA following a RP for prostate cancer at Mayo Clinic in Jacksonville, Florida, or Rochester, Minnesota, between July 1987 and February 2009 (*N* = 403). From this initial cohort, we selected all patients who had serum calcium and serum albumin measurements taken simultaneously after RP and within 12 months before the initiation of SRT, resulting in a final sample size of 165 patients who were included in the main analysis of this study. These patients received SRT between July 1987 and November 2008. Median time from serum calcium and serum albumin measurements to SRT initiation was 20 days (range: 1–362 days), and median time from RP to initiation of SRT was 22.0 months (range: 0.2–181.5 months). Corrected calcium was used in all analyses and was calculated as follows: corrected calcium = serum calcium + (0.8 × [4.0 − serum  albumin]).

The temporal relationship between calcium measurement and dietary intake or hormone therapy was unknown. When we compared the 165 patients who were included in this study with the remaining 238 patients from the original cohort who did not meet our inclusion criteria, we noted no significant differences (all *P* ≥ 0.088) except that the 238 patients who were excluded from the main analysis received slightly higher SRT doses than the 165 included patients (median: 66.6 Gy versus 65.1 Gy, *P* = 0.004). Of note, the estimated cumulative incidences of BCR at 3 and 5 years after SRT initiation were very similar between the 165 included and 238 excluded patients (42% versus 41% and 52% versus 51%, resp., *P* = 0.36). We defined BCR after SRT as a single PSA value of 0.4 ng/mL or higher, which had exceeded the post-SRT nadir, as described by Amling et al. [10].

### 2.2. Salvage Radiation Therapy Information

Patients were treated with 6 to 20 MV photons. The target volume was defined as the prostatic fossa with or without the seminal vesicles. The prostatic fossa was contoured on the basis of the estimated preoperative location of the prostate. Computed-tomography-based treatment planning or standard radiographic landmarks with or without surgical clip location were used to define the treatment volume. Contrast media were placed in the bladder and rectum at the time of simulation, and retrograde urethrography was performed to aid in the identification of the prostatic fossa and for partial shielding of these normal organs. The treatment technique evolved during the study period from 2-dimensional, multiple-field blocking to 3-dimensional conformal to intensity-modulated radiation therapy with 5 or 9 coplanar beams. Image-guided techniques were used in the latter part of the study period. A median dose of 65.0 Gy (range: 54.0–72.4 Gy) was administered to the prostatic fossa in 1.8 to 2.0 Gy fractions. After the completion of SRT, patients were evaluated (medical history, physical examination, and serum PSA measurements) every 3 to 6 months for 5 years and yearly thereafter.

### 2.3. Statistical Analysis

Continuous variables were summarized with the sample median, minimum, and maximum. Categorical variables were summarized with number and percentage. The Kaplan-Meier method was used to estimate the cumulative incidence of BCR after SRT initiation, censoring at the date of last followup. Cox proportional hazards models were used to evaluate the association between serum calcium and BCR; relative risks (RRs) and 95% confidence intervals (CIs) were estimated. Single variable models were utilized as well as multivariable models where we adjusted for factors (pathological tumor stage, Gleason score, pre-SRT PSA, and SRT dose) that have previously associated with BCR in the overall patient cohort [2, 11]. Sensitivity of results to additional individual adjustment for other factors was also examined. We considered pre-SRT calcium as a continuous variable to evaluate a linear association with BCR and also as a 3-level categorical variable based on the approximate sample tertiles in order to evaluate a possible nonlinear association. *P* values of 0.05 or less were considered as statistically significant. Statistical analyses were performed using SAS software (SAS Institute, Cary, NC) and R Statistical Software (version 2.14.0; R Foundation for Statistical Computing, Vienna, Austria).

## 3. Results

Median pre-SRT serum calcium level was 9.18 mg/dL (range: 8.18–10.38 mg/dL). Our cohort of 165 patients was divided into 3 groups on the basis of approximate sample tertiles of serum calcium. There were 49 patients with low serum calcium (≤9.0 mg/dL), 59 patients with moderate serum calcium (>9.0 mg/dL and ≤9.35 mg/dL), and 57 patients with high serum calcium (>9.35 mg/dL). A comparison of characteristics of these 3 groups is shown in Table 1, where there are no noticeable differences between groups (all *P* ≥ 0.12). Of note, the time from serum calcium measurement to SRT initiation was similar in the low, moderate, and high serum calcium groups (*P* = 0.73).

**Figure 1:**
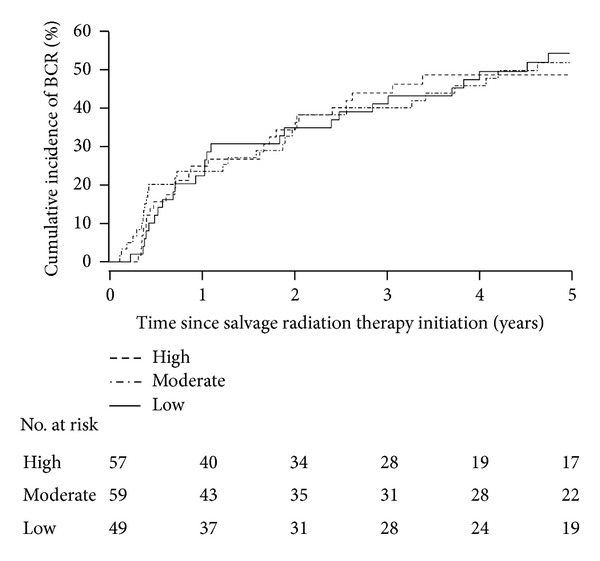
Cumulative incidence of biochemical recurrence (BCR) of prostate cancer after salvage radiation therapy (SRT) for patients with low (≤9.0 mg/dL), moderate (>9.0 and ≤9.35 mg/dL), and high (>9.35 mg/dL) pre-SRT serum calcium levels.

The median duration of followup after the start of SRT was 37 months (range: 2–170 months), with 103 patients (62%) experiencing BCR. Estimated cumulative incidences of BCR at 3 and 5 years after SRT initiation for the entire cohort were 42% (95% CI: 34%–49%) and 52% (95% CI: 43%–59%), respectively; these cumulative incidences are displayed separately for patients with low, moderate, and high pre-SRT serum calcium levels in Figure 1. A summary of the results of single variable association analysis regarding serum calcium and BCR is provided in Table 2. Without adjustment for any potentially confounding variables, there was no evidence of a linear association between pre-SRT serum calcium and BCR (RR: 0.96 [per 0.5 mg/dL increase], 95% CI: 0.74–1.25, *P* = 0.76). Similarly, there was no evidence of an overall difference in BCR between patients with low, moderate, and high pre-SRT serum calcium levels (*P* = 0.85). More specifically, compared with patients in the low serum calcium group, there was no statistically significant difference in the risk of BCR for patients with moderate serum calcium (RR: 0.94, 95% CI: 0.59–1.50, *P* = 0.79) or high serum calcium (RR: 1.08, 95% CI: 0.66–1.75, *P* = 0.76).

Results of the multivariable Cox regression analysis evaluating the association between pre-SRT serum calcium and BCR are shown in Table 3. When considering serum calcium as a continuous variable and adjusting for pathological tumor stage, Gleason score, pre-SRT PSA, and SRT dose, we still observed no association between serum calcium and BCR (RR: 0.95 [per 0.5 mg/dL increase], 95% CI: 0.71–1.28, *P* = 0.75). Similarly, when adjusting for these 4 risk factors for BCR, the lack of difference in risk of BCR between patients with low, moderate, and high serum calcium levels also remained unchanged; in comparison to patients with low serum calcium, the risk of BCR was not significantly different for patients with moderate (RR: 0.91, 95% CI: 0.55–1.48, *P* = 0.69) or high (RR: 1.21, 95% CI: 0.72–2.04, *P* = 0.47) serum calcium levels. Additional individual adjustment for other variables, such as time from serum calcium measurement to SRT initiation and year of SRT initiation, did not alter these results (Table 3).

In further examination of the association between pre-SRT serum calcium and BCR, we performed 2 separate sensitivity analyses regarding the 12-month pre-SRT initiation cutoff, still requiring that all serum calcium and albumin measurements be taken simultaneously and after RP. In the first sensitivity analysis, we considered only patients who had serum calcium and albumin measurements taken within 3 months before SRT. In this cohort of 123 patients, there was no evidence of a linear relationship between serum calcium and BCR (RR: 1.01, 95% CI: 0.71–1.45, *P* = 0.95) in multivariable analysis adjusting for pathological tumor stage, Gleason score, pre-SRT PSA, and SRT dose nor was there evidence of a difference in the risk of BCR between patients with low, moderate, and high calcium levels (RR (moderate versus low): 0.80, RR (high versus low): 1.32, and overall *P* = 0.21). In our second sensitivity analysis, we expanded our inclusion criteria and utilized all 196 patients who had serum calcium and albumin measurements taken at any time before SRT but after RP again provided that serum calcium and albumin were measured simultaneously. In this larger patient group, there was again no significant linear association between serum calcium and BCR (RR: 0.98, 95% CI: 0.73–1.30, *P* = 0.86) in multivariable analysis and also no difference in the risk of BCR between patients with low, moderate, and high calcium levels (RR (moderate versus low): 0.88, RR (high versus low): 1.20, overall *P* = 0.46).

## 4. Discussion

The ability to appropriately select men who are candidates for SRT following BCR after RP remains a key issue in the treatment of prostate cancer. Ultimately, the question centers on the ability to distinguish more accurately men with a detectable PSA after RP with a local recurrence from men with micrometastatic disease. While we have previously published a scoring algorithm that assists in stratifying men into low, moderate, and high risk of BCR after SRT, we are mindful of the need to explore additional means of enhancing prognostication for men considering SRT. In addition, we should seek to prioritize our efforts to examine patient-related features that are easily obtainable through routine examination so potential prognostic benefits are costeffective and utilize limited resources well. Previous investigators provided evidence linking serum calcium level to adverse outcomes in men with prostate cancer. Herein, we extend these findings by examining the association of serum calcium and risk of BCR in men undergoing SRT.

Human prostate cancer cell lines express both calcium-sensing receptors (CaRs) and parathyroid hormone-related protein (PTHrP) receptors [12, 13]. Increased levels of calcium stimulate release of PTHrP via CaRs [12]. PTHrP and parathyroid hormone (PTH) bind to PTHrP receptors with equal affinity. Increased proliferation and chemotaxis of prostate cancer cell lines have been linked with binding of PTH to these receptors [13]. These data suggest that increased serum calcium levels initiate a feedback loop through which prostate cancer cells proliferate and metastasize. This relationship was validated in a study that revealed elevated calcium promoted proliferation and enhanced attachment of cells in prostate cancer cell lines [14]. The discovery of these mechanisms prompted investigators to evaluate for any association between increased calcium and prostate cancer outcomes.

Several investigators studied the association of dietary calcium intake and prostate cancer, but the findings were inconsistent [15, 16]. Such an inconsistency is not unexpected as changes in dietary calcium are unlikely to result in significant variation of serum calcium due to tight homeostatic control. Therefore, serum calcium may be a more appropriate marker for study.

Skinner and Schwartz [8] evaluated the association of serum calcium and prostate cancer incidence and mortality in a prospective cohort of men involved in the NHANES I and NHANES Epidemiologic Followup Study. With 2,814 men and 46,188 person-years of followup, 85 cases of prostate cancer and 25 prostate cancer deaths occurred. While an association between serum calcium and prostate cancer incidence was not observed, men with a serum calcium in the highest tertile (mean serum calcium, 10.2 ± 0.3 mg/dL) had a significantly higher risk of prostate cancer mortality compared with men in the lowest tertile (mean serum calcium, 9.3 ± 0.3 mg/dL), even after adjustment for age, body mass index, race, and family history (RR: 2.59, 2.62, 2.68, and 2.68, resp.). A Swedish prospective cohort study [17] of 22,391 men also found no association of increased serum calcium with prostate cancer incidence, but the study did not evaluate prostate cancer mortality.

Total serum calcium includes both physiologically active ionized calcium and bound calcium. As a result, total serum calcium varies with changes in albumin concentration, whereas ionized calcium has minimal variation and may be a more reliable marker. In a follow-up study, Skinner and Schwartz [9] evaluated the association of total and ionized calcium with prostate cancer mortality in a prospective cohort of men from NHANES III. Twenty-five prostate cancer deaths occurred in the cohort of 6,710 men with 56,625 person-years of follow-up. Compared with men in the lowest tertile of total serum calcium (mean total calcium, 8.80 mg/dL [2.20 mmol/L]), men in the highest tertile (mean total calcium, 9.68 mg/dL [2.42 mmol/L]) had a multivariate adjusted RR of prostate cancer death of 2.07. Similarly, men with the highest tertile of ionized calcium had an RR of 3.18 for prostate cancer death.

Subsequently, researchers at the Mayo Clinic identified 7,650 patients with serum calcium measurements taken prior to RP for localized prostate cancer [18]. Serum calcium was analyzed categorically as below normal (<8.9 mg/dL), normal (8.9–10.1 mg/dL), and above normal (>10.1 mg/dL) levels. On multivariate analysis, increased calcium was not significantly associated with BCR, local recurrence, systemic progression, or cancer-specific death.

In our cohort of 165 patients, we did not observe an association between serum calcium and BCR following SRT. As with all studies in which a statistical association is not identified, it is important to note that we cannot conclude definitively that no such association exists. However, based on the 95% CIs for estimated RRs, we can reasonably state that any association between serum calcium and BCR is likely small to moderate and therefore unlikely to be clinically meaningful. Given this, serum calcium does not appear to be a promising factor for prediction of BCR following SRT.

The median age in our study was higher compared with that of men in NHANES. Because younger age is associated with higher calcium values, our overall distribution of calcium values was lower such that our highest calcium tertile was a calcium level greater than 9.35 mg/dL. Therefore, it is possible that our patients did not express the high normal calcium levels found to be predictive of prostate cancer mortality in the NHANES study.

We acknowledge the limitations of our study. As previously referred to, with a relatively small sample size of 165 patients of whom 103 experienced BCR after SRT, power-to-detect associations between serum calcium and BCR were limited, and the possibility of type II error (i.e., a false-negative association) is important to consider. Our study is also limited by its retrospective nature. The measurement of serum calcium and albumin was not standardized and varied between patients regarding the length of time from this measurement to the start of SRT. However, this is unlikely to have affected our results as the time interval between serum calcium and albumin measurements to initiation of SRT did not differ significantly between the 3 calcium groups, and the lack of association between pre-SRT serum calcium and BCR was consistent when adjusting for this factor in multivariable analysis. Due to the lack of pre-SRT ionized calcium determinations in our cohort, we analyzed total serum calcium instead. We did adjust for serum albumin, thus correcting in part for this deficiency.

## 5. Conclusions

We did not observe an association between serum calcium and increased risk of BCR in our cohort of men treated with SRT for a detectable PSA after RP. Therefore, pretreatment calcium determination is unlikely to be a useful tool in predicting the risk of BCR following SRT.

## Figures and Tables

**Table 1:** Patient characteristics according to pre-SRT serum calcium level.

Variable	Pre-SRT serum calcium level			*P* value
	Low: ≤9.0 mg/dL (*N* = 49)	Moderate: >9.0 and ≤9.35 mg/dL (*N* = 59)	High: >9.35 mg/dL (*N* = 57)	
Pre-RP PSA level (ng/mL)	11.0 (3.4, 32.5)	11.1 (2.6, 56.7)	7.3 (2.0, 44.4)	0.29
Pre-SRT PSA level (ng/mL)	0.7 (0.2, 15.3)	0.6 (0.2, 36.1)	0.8 (0.1, 4.9)	0.12
SRT dose (Gy)	64.8 (60.0, 70.2)	64.8 (58.4, 70.2)	66.6 (54.0, 72.4)	0.16
Age at SRT initiation	67 (48, 80)	68 (55, 79)	68 (44, 85)	0.38
Time from RP to SRT initiation (months)	22 (<1, 117)	20 (2, 126)	24 (3, 181)	0.50
Time from serum calcium measurement to SRT initiation (days)	16 (1, 362)	21 (<1, 361)	21 (<1, 362)	0.73
Pathological tumor stage				0.68
T2	22 (45%)	24 (41%)	28 (49%)	
T3a	18 (37%)	20 (34%)	16 (28%)	
T3b	9 (18%)	15 (25%)	13 (23%)	
Surgical margin				0.66
Positive	27 (57%)	34 (58%)	37 (65%)	
Negative	20 (43%)	25 (42%)	20 (35%)	
Gleason score				0.49
3–6	17 (38%)	18 (31%)	22 (39%)	
7	19 (42%)	26 (45%)	25 (45%)	
8–10	9 (20%)	14 (24%)	9 (16%)	
Pre-SRT hormone therapy				0.81
Yes	7 (14%)	10 (17%)	11 (19%)	
No	42 (86%)	49 (83%)	46 (81%)	
Year of start of SRT				0.29
1987–1992	12 (24%)	16 (27%)	14 (25%)	
1993–1997	18 (37%)	13 (22%)	12 (21%)	
1998–2001	16 (33%)	14 (24%)	10 (18%)	
2002–2008	3 (6%)	16 (27%)	21 (37%)	

The sample median (minimum and maximum) is given for continuous variables. *P* values result from Fisher's exact test or a Kruskal-Wallis rank sum test. Information was not available for the following variables: preoperative PSA (low: 7, moderate: 2, and high: 6), surgical margin (low: 2), and Gleason score (Low: 4, Moderate: 1, High: 1). RP: radical prostatectomy. PSA: prostate specific antigen. SRT: salvage radiation therapy.

**Table 2:** Single variable analysis association between pre-SRT serum calcium and BCR of prostate cancer after SRT.

Serum calcium	Cumulative incidence of BCR (95% CI)			*P* value
	3 years after SRT	5 years after SRT	RR (95% CI)	
Continuous variable (per 0.5 mg/dL increase)	N/A	N/A	0.96 (0.74, 1.25)	0.76
Categorical variable				
Low: ≤9.0 mg/dL	41% (26%–54%)	55% (38%–67%)	1.00 (reference)	N/A
Moderate: >9.0 and ≤9.35 mg/dL	40% (26%–52%)	52% (37%–64%)	0.94 (0.59, 1.50)	0.79
High: >9.35 mg/dL	44% (29%–56%)	49% (33%–62%)	1.08 (0.66, 1.75)	0.76

Relative risks, 95% confidence intervals, and *P* values result from single variable the Cox proportional hazards regression models. BCR: biochemical recurrence. RR: relative risk. CI: confidence interval. SRT: salvage radiation therapy. N/A: not applicable.

**Table 3:** Multivariable analysis association between pre-SRT serum calcium and BCR of prostate cancer after SRT.

Model adjustment	Association of pre-SRT serum calcium and BCR of prostate cancer after SRT					
	Pre-SRT serum calcium as a continuous variable (0.5 mg/dL increase)		Moderate versus low pre-SRT serum calcium		High versus low pre-SRT serum calcium	
	RR (95% CI)	*P* value	RR (95% CI)	*P* value	RR (95% CI)	*P* value
No adjustment for other variables	0.96 (0.74, 1.25)	0.76	0.94 (0.59, 1.50)	0.79	1.08 (0.66, 1.75)	0.76
Adjusting individually for risk factors for BCR						
Pathologic tumor stage	0.99 (0.75, 1.30)	0.91	0.89 (0.55, 1.42)	0.61	1.12 (0.69, 1.82)	0.65
Gleason score	0.94 (0.74, 1.25)	0.68	0.96 (0.59, 1.56)	0.86	1.15 (0.69, 1.92)	0.59
Pre-SRT PSA	0.95 (0.73, 1.24)	0.70	0.93 (0.58, 1.49)	0.76	1.10 (0.68, 1.78)	0.71
SRT dose	0.98 (0.74, 1.28)	0.86	0.91 (0.57, 1.45)	0.68	1.10 (0.68, 1.79)	0.70
Adjusting for all four risk factors for BCR	0.95 (0.71, 1.28)	0.75	0.91 (0.55, 1.48)	0.69	1.21 (0.72, 2.04)	0.47
Adjusting for all four risk factors for BCR and:						
Preoperative PSA	0.93 (0.67, 1.28)	0.65	0.88 (0.53, 1.47)	0.63	1.11 (0.64, 1.94)	0.72
Age at SRT initiation	0.98 (0.72, 1.32)	0.87	0.93 (0.57, 1.52)	0.76	1.26 (0.74, 2.13)	0.39
Time from RP to SRT initiation	0.96 (0.71, 1.29)	0.76	0.90 (0.55, 1.48)	0.69	1.24 (0.73, 2.09)	0.43
Time from serum calcium measurement to SRT initiation	0.95 (0.70, 1.28)	0.74	0.89 (0.54, 1.45)	0.64	1.21 (0.72, 2.03)	0.48
Surgical margin	0.93 (0.69, 1.26)	0.63	0.88 (0.54, 1.44)	0.61	1.16 (0.69, 1.96)	0.57
Pre-SRT hormone therapy	0.94 (0.70, 1.26)	0.66	0.86 (0.53, 1.40)	0.55	1.20 (0.72, 2.02)	0.48
Year of start of SRT	0.95 (0.70, 1.28)	0.73	0.90 (0.55, 1.47)	0.67	1.19 (0.70, 2.01)	0.51

Relative risks, 95% confidence intervals, and *P* values result from the Cox proportional hazards regression models. Low pre-SRT serum calcium was considered to be ≤9.0 mg/dL. Moderate pre-SRT serum calcium was considered to be >9.0 mg/dL and ≤9.35 mg/dL. High pre-SRT serum calcium was considered to be >9.35 mg/dL. BCR: biochemical recurrence. RR: relative risk. CI: confidence interval. SRT: salvage radiation therapy. RP: radical prostatectomy.
